# Author Correction: Spatial modeling algorithms for reactions and transport in biological cells

**DOI:** 10.1038/s43588-025-00773-1

**Published:** 2025-02-03

**Authors:** Emmet A. Francis, Justin G. Laughlin, Jørgen S. Dokken, Henrik N. T. Finsberg, Christopher T. Lee, Marie E. Rognes, Padmini Rangamani

**Affiliations:** 1 Department of Pharmacology, University of California San Diego School of Medicine, La Jolla, CA USA; 2 Department of Mechanical and Aerospace Engineering, University of California San Diego, La Jolla, CA USA; 3 Computational Engineering Division, Lawrence Livermore National Laboratory, Livermore, CA USA; 4 Department of Numerical Analysis and Scientific Computing, Simula Research Laboratory, Oslo, Norway; 5 Department of Computational Physiology, Simula Research Laboratory, Oslo, Norway; 6 Department of Molecular Biology, University of California San Diego, La Jolla, CA USA; 7 K. G. Jebsen Centre for Brain Fluid Research, University of Oslo, Oslo, Norway

**Keywords:** Computer modelling, Cellular signalling networks, Software, Numerical simulations, Software

Correction to: *Nature Computational Science* 10.1038/s43588-024-00745-x, published online 19 December 2024.

In the version of this article initially published, there was an error in Fig. 3a, where the red inhibition arrow from “LIMK” to “Cofilin” was originally a green right-facing arrow. The figure is amended in the HTML and PDF versions of the article.

