# Supplementary material for: Spatial modeling algorithms for reactions and transport in biological cells
Source: Nat Comput Sci. 2024 Dec 19;5(1):76–89. doi: 10.1038/s43588-024-00745-x (PMC11774757; doi:10.1038/s43588-024-00745-x)
Supplement: Supplementary file 1 — Supplementary Notes 1–5, Tables 1–26, Figs. 1–3, captions for Videos 1–7 and References. [file 43588_2024_745_MOESM1_ESM.pdf]

nature computational science

Supplementary information

https://doi.org/10.1038/s43588-024-00745-x

Spatial modeling algorithms for reactions and transport in biological cells

In the format provided by the authors and unedited

Supplementary Information

Supplementary Note 1: A general framework for multi-domain and multi-species reaction and transport

SMART is designed to represent and solve coupled systems of nonlinear ordinary and partial differential equations, describing reactions and/or transport due to diffusion, convection or drift in multi-domain geometries including subdomains of co-dimension zero or one (volume-surface problems). This section defines the range and scope of SMART by describing its foundational mathematical modelling framework. SMART uses a model specification framework inspired by the Systems Biology Markup Language[1], consisting of compartments, species, reactions, and parameters. Throughout this mathematical overview, we refer to objects within SMART that correspond to different terms in the equations.

1.1 Notation

- $\mathcal{M}$: index/label set for domains Ω^m ($m \in \mathcal{M}$).
- $\mathcal{Q}$: index/label set for the surfaces Γ^q ($q \in \mathcal{Q}$).
- $\mathcal{I}$, $\mathcal{I}^m$, $\mathcal{I}^q$: index/label sets for the species, the species in Ω^m and species on Γ^q, respectively.
- $\mathcal{K}$, $\mathcal{K}^m$, $\mathcal{K}^q, \mathcal{K}^{mq}, \mathcal{K}^{mqn}$: index/label sets for the reactions, reactions in Ω^m, reactions on Γ^q, volume-surface reactions between species in Ω^m and Γ^q, and volume-surface-volume reactions between species in Ω^m, Γ^q, and Ω^n.

1.2 Multi-domain and multi-surface geometry representation

We consider an open topologically D-dimensional manifold $\Omega \subset \mathbb{R}^d$ for $D \leqslant d = 1, 2, 3$, and assume that Ω is partitioned into $|\mathcal{M}|$ open and disjoint *domains* $\Omega^m \subset \mathbb{R}^d$:

$$\Omega = \bigcup_{m \in \mathcal{M}} \Omega^m, \tag{1}$$

each with (internal or external) boundary $\partial\Omega^m$, and boundary $\partial\Omega$. In the SMART framework, each domain Ω^m is referred to as a volume compartment. The *boundary normal* $\mathbf{n}^m$ on $\partial\Omega^m$ is the outward pointing normal vector field to the boundary. The interface Γ^{mn} between domains Ω^m and Ω^n is defined as the intersection of the closure of the domains:

$$\Gamma^{mn} = \overline{\Omega^m} \cap \overline{\Omega^n}, \tag{2}$$

for $m, n \in \mathcal{M}$, and may or may not be empty. Each interface Γ^{mn} may be further partitioned into one or more *surfaces*:

$$\Gamma^{mn} = \bigcup_{q \in \mathcal{Q}^{mn}} \Gamma^q, \tag{3}$$

where each Γ^q is referred to as a surface compartment within SMART. We denote the total set of surfaces by $\mathcal{Q} = \cup_{m,n \in \mathcal{M}} \cup \mathcal{Q}_0$ where $\mathcal{Q}_0$ also includes (sub)surfaces on the boundary of Ω. For each surface, we define the surface-to-neighbors map from the surface index q to its neighboring domain indices m, n: $q \mapsto m, n$. If the surface is a real boundary surface, the map returns the single neighbor $q \mapsto m,$.

Moreover, let time $t \in [0, T]$ for $T > 0$.

1.3 Coupled multi-domain reaction-transport equations

Different physical processes may occur on domains or on surfaces. For instance, in the context of computational neuroscience at the cellular level, different species and processes dominate in the dendrites and axons, and on the plasma membrane versus at post-synaptic densities. In general, SMART is designed to model different species coexisting, interacting and moving within a (sub)domain or (sub)surface, between (sub)domains and across (sub)surfaces. Within the SMART framework, information about each species is stored within an object, and the equations describing their interactions are stored within reaction objects. Any other quantities involved in the reaction equations (e.g., reaction rates and binding affinities) are defined separately and stored within parameter objects.

Domain species

We define and denote the set of species coexisting in Ω^m by $\mathcal{I}^m$ for each (sub)domain Ω^m. For each m, the species $i \in \mathcal{I}^m$ with concentrations $u_i^m = u_i^m(x,t)$ for $x \in \Omega^m$ and $t \in (0,T]$ satisfy reaction-transport equations of the form

$$\partial_t u_i^m + \mathcal{T}_i^m(u_i^m) - f_i^m(u^m) = 0 \qquad \text{in } \Omega^m, \tag{4}$$

where ∂_t is the time-derivative, $\mathcal{T}_i^m$ defines transport terms, and f_i^m are volume reactions i.e. reactions within the domain, typically given by non-linear and non-trivial relations involving one or more of the species $u^m = \{u_i^m\}_{i \in \mathcal{I}^m}$. In the case of transport by diffusion alone,

$$\mathcal{T}_i^m(u) = -\nabla \cdot (D_i^m \nabla(u)), \tag{5}$$

where $\nabla\cdot$ is the spatial divergence operator, ∇ is the spatial gradient, and D_i^m is the diffusion coefficient of species i in domain Ω^m which may be heterogeneous i.e., spatially varying and/or anisotropic i.e., tensor-valued. Remaining relative to Ω^m, on (all) surfaces $\Gamma^q \subseteq \Gamma^{mn}$, we assume that the flux of species i is governed by a relation of the form:

$$D_i \nabla u_i^m \cdot \mathbf{n}^m - R_i^q(u^m, u^n, v^q) = 0 \qquad \text{on } \Gamma^q, \tag{6}$$

where R_i^q defines surface fluxes, and the surface concentrations v^q are defined below.

Surface species

The surface concentrations $v^q = \{v_j^q\}_{j \in \mathcal{I}_q}$, entering in Supplementary Equation (6) above, are defined on $\Gamma^q \subseteq \Gamma^{mn}$, either as prescribed fields or via surface equations as follows: find $v^q = v^q(x,t)$ for $x \in \Gamma^q$, $t \in (0,T]$ such that

$$\partial_t v_j^q + \mathcal{T}_i^q(v_i^q) - g_j^q(u^m, u^n, v^q) = 0 \qquad \text{on } \Gamma^q, \tag{7}$$

where g_j^q are surface reactions for each species $j \in \mathcal{I}^q$. In the case of transport by surface diffusion, with surface diffusion coefficient D_j^q for species j, surface gradient ∇_S and surface divergence $\nabla_S\cdot$, we have:

$$\mathcal{T}_j^q(v) = -\nabla_S \cdot (D_j^q \nabla_S v). \tag{8}$$

Boundary conditions

For any (sub)boundary $\Gamma^q \subset \partial\Omega^m$ such that $\Gamma^q \subseteq \partial\Omega$, the following boundary conditions are prescribed:

$$D_i^m \nabla u_i^m \cdot \mathbf{n}^m - R_i^q(u^m, v^q) = 0, \tag{9}$$

which can be seen as a special case of Supplementary Equation (6) in which there is no adjacent volume compartment. For any species v^q on any surface Γ^q with non-empty boundary, zero flux boundary conditions are prescribed:

$$D_j^q \nabla_S v_j^q \cdot \mathbf{n}^q = 0, \tag{10}$$

where $\mathbf{n}^q$ is the normal to the boundary line.

Initial conditions

Initial conditions are required for any u_i^m for $m \in \mathcal{M}$, $i \in \mathcal{I}^m$, and v_i^q for $q \in \mathcal{Q}$, $i \in \mathcal{I}^q$.

1.4 SMART model generation from reaction specifications

Here, we outline the conventions used internally by SMART to convert reaction specifications into associated terms in PDEs and boundary conditions. In SMART, each reaction has several "flux" objects associated with it - one for every reactant and every product. The manner in which these fluxes are computed depends on the type of reaction, generally classified as "volume", "surface", or "volume-surface", or "volume-surface-volume" (Figure 2 in the main text).

Considering the set of all expressions associated with volume reactions within compartment Ω^m, $\mathcal{F}^m = \{\mathcal{F}_k^m\}_{k \in \mathcal{K}^m}$, the flux associated with a reactant or product species i for reaction k is given by:

$$f_{i,k}^m = \mathcal{S}_{i,k}^m \mathcal{F}_k^m, \tag{11}$$

where $\mathcal{S}_{i,k}^{m}$ is the stoichiometric coefficient, which is positive for products and negative for reactants and whose magnitude matches the number of molecules generated or consumed.

Surface reactions are handled analogously. The set of all surface reactions within compartment Γ^q is $\mathcal{G}^q = \{\mathcal{G}_k^q\}_{k\in\mathcal{K}^q}$, and the flux associated with a reactant or product species j for reaction k is given by:

$$g_{j,k}^{q} = \mathcal{S}_{j,k}^{q}\mathcal{G}_k^q, \tag{12}$$

where $\mathcal{S}_{i,k}^{m}$ is the stoichiometric coefficient as before.

Volume-surface reactions generally contribute to both PDEs and boundary conditions as follows. Considering the set of all expressions associated with volume-surface reaction k between compartments Ω^m and Γ^q, $\mathcal{R}^{mq} = \{\mathcal{R}_k^{mq}\}_{k\in\mathcal{K}^{mq}}$, the flux associated with a reactant or product volume species i for reaction k is given by:

$$R_{i,k}^{mq} = \alpha_{i,k}^{mq}\mathcal{S}_{i,k}^{mq}\mathcal{R}_k^{mq}, \tag{13}$$

where $\mathcal{S}_{i,k}^{m}$ is the stoichiometric coefficient and $\alpha_{i,k}^{mq}$ is a scaling factor converting molecular flux (molecules per unit surface per unit time) into the dimensionally equivalent units of volume concentration multiplied by length per unit time. For any surface species j that acts as a product or reactant, such reactions have an associated flux:

$$g_{j,k}^{mq} = \mathcal{S}_{j,k}^{mq}\mathcal{R}_k^{mq}, \tag{14}$$

where $\mathcal{S}_{j,k}^{mq}$ is the stoichiometric coefficient.

The remaining case of a surface-mediated reaction between two adjacent volume compartments ("volume-surface-volume" reaction) is a natural extension of the above "volume-surface" case. The set of expressions for all such reactions between species in Ω^m, Γ^q, and Ω^n is $\mathcal{R}^{mqn} = \{\mathcal{R}_k^{mqn}\}_{k\in\mathcal{K}^{mqn}}$ and the contribution to reactants and products are given by replacing all cases of mq with mqn in Supplementary Equations (13) and (14).

All of these fluxes are included in Supplementary Equations (4), (6), (7) and (9) by summing the associated contributions for a given species. In particular, the total reaction term f_i^m in Supplementary Equation (4) is given by summing the contribution from all volume reactions involving species i, $\mathcal{K}_i^m$:

$$f_i^m = \sum_{k\in\mathcal{K}_i^m} f_{i,k}^m. \tag{15}$$

The total reaction term R_i^q in Supplementary Equation (6) or Supplementary Equation (9) is given by summing the contribution from all volume-surface or volume-surface-volume reactions involving species i ($\mathcal{K}_i^{mq}$, $\mathcal{K}_i^{mqn}$):

$$R_i^q = \sum_{k\in\mathcal{K}_i^{mq}} R_{i,k}^{mq} + \sum_{k\in\mathcal{K}_i^{mqn}} R_{i,k}^{mqn}, \tag{16}$$

where the second term is equal to zero when applied to Supplementary Equation (9), where there is no adjacent volume compartment.

Finally, the total reaction term g_j^q in Supplementary Equation (7) is given by summing the contribution from all surface reactions involving species j, $\mathcal{K}_j^q$ as well as any volume-surface or volume-surface-volume reactions involving species j ($\mathcal{K}_j^{mq}$, $\mathcal{K}_j^{mqn}$):

$$g_j^q = \sum_{k\in\mathcal{K}_j^q} g_{j,k}^q + \sum_{k\in\mathcal{K}_j^{mq}} g_{j,k}^{mq} + \sum_{k\in\mathcal{K}_j^{mqn}} g_{j,k}^{mqn}. \tag{17}$$

Example of assembly from reactions

As a simple case of model generation in SMART, we consider the bulk-surface reaction from Rangamani et al.[2], which is also included as Example 2 in the SMART documentation. In brief, this model involves a volume species A in the cytosolic domain Ω_{cyto} that can bind to the surface species X on the plasma membrane domain Γ_{PM} to form surface species B (Supplementary Figure 3A). Treating A and X as reactants and B as a product, the rate of this volume-surface reaction is defined according to mass conservation:

$$\mathcal{R}_1^{cyto\text{-}PM} = k_{on}u_A v_X - k_{off}v_B \tag{18}$$

From Supplementary Equation (13), the boundary flux for species A is then:

$$R_{A,1}^{cyto\text{-}PM} = -\alpha_{A,1}^{cyto\text{-}PM}\mathcal{R}_1^{cyto\text{-}PM}, \tag{19}$$

where $\alpha_{A,1}^{cyto\text{-}PM}$ depends on the chosen units. For instance, in a 3D/2D system with volume concentration units of μM and surface concentration units of molecules/μm ^{2}, $\alpha_{A,1}^{cyto\text{-}PM} = \frac{1\times10^{21}}{N_A}$ μM μm^{-3}, where N_A is Avogadro's number. From Supplementary Equation (14), the surface reaction rate for X is:

$$g_{X,1}^{cyto\text{-}PM} = -\mathcal{R}_1^{cyto\text{-}PM}, \tag{20}$$

and the surface reaction rate for B is:

$$g_{B,1}^{cyto\text{-}PM} = \mathcal{R}_1^{cyto\text{-}PM}. \tag{21}$$

In terms of these expressions, the system of PDEs is:

$$\frac{\partial u_A}{\partial t} = D_A \nabla^2 u_A \quad \text{in } \Omega_{cyto} \tag{22}$$

$$D_A(\mathbf{n} \cdot \nabla u_A) = R_{A,1}^{cyto\text{-}PM} \quad \text{on } \Gamma_{PM} \tag{23}$$

$$\frac{\partial v_X}{\partial t} = D_X \nabla^2 v_X + g_{X,1}^{cyto\text{-}PM} \quad \text{on } \Gamma_{PM} \tag{24}$$

$$\frac{\partial v_B}{\partial t} = D_B \nabla^2 v_B + g_{B,1}^{cyto\text{-}PM} \quad \text{on } \Gamma_{PM}. \tag{25}$$

Supplementary Note 2: Numerical approximation and computational strategy

SMART solves the multi-domain reaction-transport equations outlined in Supplementary Note 1 via finite difference discretizations in time and a finite element discretization in space, using a *monolithic* approach to address the system couplings. We describe this monolithic approach here, considering linear diffusion-only transport relations for concreteness.

2.1 Monolithic solution of the multi-domain reaction-diffusion equations

Formally, we consider the Sobolev spaces $H^1(\Omega^m)$, $m \in \mathcal{M}$ of square-integrable functions on Ω^m with square-integrable weak derivatives, and analogously for $H^1(\Gamma^q)$, $q \in Q$, as well as the vector function spaces $H^1(\Omega^m, \mathbb{R}^d) = H^1(\Omega)^d$. For solving the multi-domain reaction-diffusion equations, we introduce two product spaces U and V consisting of bulk fields and surface fields, respectively:

$$U = \bigotimes_{m\in\mathcal{M}} H^1(\Omega^m; \mathbb{R}^{|\mathcal{I}^m|}), \quad V = \bigotimes_{q\in\mathcal{Q}} H^1(\Gamma^q; \mathbb{R}^{|\mathcal{I}^q|}). \tag{26}$$

To represent the solution fields u, v comprised of the separate bulk and surface components for the different species, we label

$$u = \{u^m\}_{m\in\mathcal{M}} = \{\{u_i^m\}_{i\in\mathcal{I}^m}\}_{m\in\mathcal{M}}, \quad v = \{v^q\}_{q\in\mathcal{Q}} = \{\{v_i^q\}_{i\in\mathcal{I}^q}\}_{q\in\mathcal{Q}}. \tag{27}$$

By standard techniques (integrating by test functions and integrating by parts), we rephrase the multi-domain reaction-diffusion equations in variational form, resulting in the following coupled variational problem: find $u \in U$ and $v \in V$ such that for all $\phi \in U$ and $\psi \in V$:

$$F(u, v; \phi) + G(u, v; \psi) = 0, \tag{28}$$

where both forms F and G are composed of sums over domains or surfaces and species:

$$F(u, v; \phi) = \sum_{m\in\mathcal{M}} \sum_{i\in\mathcal{I}^m} F_i^m(u, v; \phi_i^m), \qquad G(u, v; \psi) = \sum_{q\in\mathcal{Q}} \sum_{i\in\mathcal{I}^q} G_i^q(u, v; \psi_i^q). \tag{29}$$

Furthermore, with the $L^2(O)$-inner product over any given domain $O \subset \Omega$ defined as

$$\langle a, b\rangle_O = \int_O a \cdot b \,\mathrm{d}x,$$

we have defined

$$F_i^m(u, v, \phi_i^m) = \langle \partial_t u_i^m, \phi_i^m\rangle_{\Omega^m} + \langle D_i^m \nabla u_i^m, \nabla \phi_i^m\rangle_{\Omega^m} - \langle f_i^m(u^m), \phi_i^m\rangle_{\Omega^m} - \sum_{q\in\mathcal{Q}^{mn}} \langle R_i^q(u^m, u^n, v^q), \phi_i^m\rangle_{\Gamma^q}, \tag{30}$$

and, for any $q \in \mathcal{Q}^{mn}$ for given Ω^m and Ω^n interfacing Ω^m via Γ^q, finally:

$$G_i^q(u, v, \psi_i^q) = \langle \partial_t v_i^q, \psi_i^q\rangle_{\Gamma^q} + \langle D_i^q \nabla_S v_i^q, \nabla_S \psi_i^q\rangle_{\Gamma^q} - \langle g_i^q(u^m, u^n, v^q), \psi_i^q\rangle_{\Gamma^q}. \tag{31}$$

2.2 Discretization in time and space

SMART discretizes in time via an implicit (first-order) Euler scheme with time steps $0 = t_0 < t_1 < \ldots < t_{n-1} < t_n = T$ with timestep $\tau_n = t_n - t_{n-1}$. To discretize in space, we employ a conforming finite element mesh $\mathcal{T}$ defined relative to the parcellation of Ω into domains and surfaces, such that $\mathcal{T}^m \subseteq \mathcal{T}$ defines a submesh of Ω^m for $m \in \mathcal{M}$, and $\mathcal{G}^q$ defines a (co-dimension one) mesh of Γ^q for $q \in \mathcal{Q}$. We define the finite element spaces of continuous piecewise linears $\mathcal{P}_1$ defined relative to each (sub)mesh, and define the product spaces

$$U_h = \bigotimes_{m \in \mathcal{M}} \mathcal{P}_1^{|\mathcal{I}_m|}(\mathcal{T}^m), \qquad V_h = \bigotimes_{q \in \mathcal{Q}} \mathcal{P}_1^{|\mathcal{I}_q|}(\mathcal{G}^q). \tag{32}$$

For each time step t_i, given discrete solutions u_h^- and v_h^- at the previous time t_{i-1}, we then solve the nonlinear, coupled time-discrete problem: find $u_h \in U_h$ and $v_h \in V_h$ such that for all $\phi \in U_h$, $\psi \in V_h$:

$$H(u_h, v_h) = F_{\tau_n}(u_h, v_h, \phi) + G_{\tau_n}(u_h, v_h, \psi) = 0, \tag{33}$$

where F_{τ_n} and G_{τ_n} are defined by the forms F and G after implicit Euler time-discretization and depend on the given u_h^- and v_h^-.

2.3 Nonlinear solution algorithms

By default, the monolithic nonlinear discrete system Supplementary Equation (33) is solved by Newton-Raphson iteration with a symbolically derived discrete Jacobian.

Supplementary Note 3: Software implementation

The SMART abstractions and algorithms are implemented via the open source and generally available FEniCS finite element software package (2022-version)[3]. FEniCS supports high-level specification of variational forms via the Unified Form Language (UFL)[4], symbolic differentiation of variational forms e.g. for derivation for Jacobians, automated assembly of general and nonlinear forms over finite element meshes, and high-performance linear algebra and nonlinear solvers via e.g. PETSc[5]. Below, we describe the abstractions used by SMART to translate user-level specifications into the variational form of the reaction-transport equations summarized above.

3.1 SMART model components

A model in SMART consists of a collection of four classes of objects - compartments, species, reactions, and parameters.

A compartment is initialized by a name, dimensionality, associated length units, and marker value in the parent mesh. Each compartment is linked to a parent mesh by marker values stored in discrete functions defined over mesh entities, M_{facet} defined over all mesh facets (faces for 2D parent mesh, edges for 3D parent mesh) and M_{cell} defined over all mesh cells. For instance, in a 3D/2D model, M_{cell} stores a marker value associated with each tetrahedron in the geometry; if the cytosol is linked to marker value "1", then the cytosol is comprised of all tetrahedra with value "1" in M_{cell}. All information about the compartment topology is implicit in the mesh functions, but evaluation can be sped up by users explicitly specifying which compartments are nonadjacent.

A species is initialized by a name, initial condition, units, diffusion coefficient, diffusion coefficient units, and compartment. Importantly, each species is associated with only one compartment; a single molecule that moves between compartments will be specified by two species (e.g., cytosolic Ca^{2+} and ER Ca^{2+}). The initial condition can be specified as a constant value or as a string giving a spatially dependent expression. The unknown symbols in an expression can include spatial coordinates x, y, z or, in the case of surface species, the curvature *curv*.

A reaction is initialized by a name, a list of reactants, a list of products, equations for the forward and reverse reaction rates, and Python dictionaries mapping strings from the equations to parameters and species initialized separately. The reactant and product lists also specify the stoichiometry of each reaction, e.g. , if a product is listed twice, it has a stoichiometric coefficient of 2. Reactions are integrated into the variational forms of each PDE as detailed above, and SMART carries out internal checks to ensure consistency in units using the Python package pint[6]. If no forward or reverse reaction strings are specified, SMART defaults to assuming mass action kinetics, with an on-rate parameter *on* and an off-rate parameter *off*. For non-mass-action kinetics, SMART supports a range of non-algebraic functions, including trigonometric functions, exponential and logarithmic functions, and absolute value

and sign functions. Furthermore, reactions can be defined as curvature sensitive by including *curv* in the reaction expression.

Finally, a parameter is initialized by a name, value, and units. The value of a parameter can be either a scalar or a string specifying a time and/or space-dependent expression, where the free symbols should only include x, y, z, t. A time-dependent parameter can also be loaded from a text file as shown in Example 5 of the SMART documentation. If a time-dependent parameter with no dependence on species concentrations is used to define a flux, the user may optionally set `pre_integration` to `True`. In this case, SMART uses a pre-integrated expression or a numerical approximation to update parameter values while ensuring consistent mass transfer; that is, given a time-dependent flux $f(t)$, flux values are updated as follows:

$$f(t+\tau_n) = \tau_n^{-1} \int_t^{t+\tau_n} f(T)dT. \tag{34}$$

When using this updating scheme together with implicit Euler time integration, the amount of mass transferred is independent of the time step.

3.2 Solver specifications and adaptive time-stepping

SMART uses PETSc4py for all linear and nonlinear solves[7]. Linear systems are solved iteratively using Krylov solvers with field-split biconjugate gradient preconditioning. The nonlinear system is solved using the Newton line search SNES solver within PETSc. Default relative tolerances in each case are set to 1×10^{-5}, but these and other solver parameters can be readily adjusted as illustrated in Example 6 of the SMART documentation.

By default, SMART takes uniform time-steps matching the initial specification. However, there is an optional function for adaptive time-stepping using the number of Newton iterations required to converge at the previous step. The rules were set *ad hoc* and are summarized in Supplementary Table 1. The reported "time-step adjustment factor" ζ is the multiplicative factor used to alter the time-step at the end of a given time step, *i.e.*, the new time-step τ_{new} is given by a in terms of the previous time-step τ_{old} as $\tau_{new} = \zeta\tau_{old}$.

Supplementary Table 1: Adaptive time-stepping rules in SMART.

Number of nonlinear iterations	Time-step adjustment factor
0-1	1.1
2-4	1.05
5-10	1.0
11-20	0.8
>20	0.5

3.3 Axisymmetric models

SMART offers the ability to assume axisymmetry given a 2D mesh. Given a 2D mesh in the $r-z$ plane, the symmetry axis is assumed to be $r = 0$. When this feature is turned on in SMART, the variational forms are adjusted accordingly, with the only necessary change being the multiplication by r in each integral (factors of 2π cancel throughout):

$$F_i^m(u, v, \phi_i^m) = \langle r\partial_t u_i^m, \phi_i^m\rangle_{\Omega^m} + \langle rD_i^m \nabla u_i^m, \nabla\phi_i^m\rangle_{\Omega^m} - \langle rf_i^m(u^m), \phi_i^m\rangle_{\Omega^m} - \sum_{q\in\mathcal{Q}^{mn}} \langle rR_i^q(u^m, u^n, v^q), \phi_i^m\rangle_{\Gamma^q} \tag{35}$$

$$G_i^q(u, v, \psi_i^q) = \langle r\partial_t v^q, \psi_i^q\rangle_{\Gamma^q} + \langle rD_i^q \nabla_S v_i^q, \nabla_S \psi_i^q\rangle_{\Gamma^q} - \langle rg_i^q(u^m, u^n, v^q), \psi_i^q\rangle_{\Gamma^q}. \tag{36}$$

In this case, the compartments must be initialized as a 2D/1D system, as shown in the mechanotransduction code and Example 3 with axisymmetry in the SMART repository.

Supplementary Note 4: Mesh generation for mechanotransduction model

Cell geometries on micropatterned substrates were generated in Gmsh[8]. As a reference geometry, we used the axisymmetric cell shape previously defined in VCell[9], where the plasma membrane surface satisfies

$$\frac{\left(1-\frac{z^4}{2000+z^4}\right)(r^2+z^2)+0.4(r^2+(z+9.72)^2)z^4}{15+z^4}=169, \tag{37}$$

and the nuclear membrane surface satisfies

$$\left(\frac{r}{5.3}\right)^2+\left(\frac{z-4.8}{2.4}\right)^2=1, \tag{38}$$

where r and z are cylindrical coordinates. These implicit boundary representations were constructed by solving for r at each z value using `solveset` in Sympy[10].

Shapes with rectangular or star-shaped contact regions were constructed by introducing an angular dependence to the reference shape. In particular, we scale the r value at the surface as a function of the cylindrical coordinate θ. Considering an implicitly (as above) or explicitly defined contour as a function of arc length $r(s), z(s)$, the scaled contour at a given value of θ is $r(s)T(\theta), z(s)$. We additionally require that this shape conserves cell volume, resulting in the constraint:

$$\int_0^{2\pi} T^2(\theta)d\theta = 2\pi. \tag{39}$$

For the circular contact region, $T(\theta)=1$ and the above constraint is automatically satisfied.

For a rectangular contact region with sides of length a and b:

$$T_{rect}(\theta)=\begin{cases}\frac{a}{2\cos\theta} & -\arctan(\frac{b}{a})<\theta\leq\arctan(\frac{b}{a})\\ \frac{b}{2\sin\theta} & \arctan(\frac{b}{a})<\theta\leq\pi-\arctan(\frac{b}{a})\\ -\frac{a}{2\cos\theta} & \pi-\arctan(\frac{b}{a})<\theta\leq\pi+\arctan(\frac{b}{a})\\ -\frac{b}{2\sin\theta} & \pi+\arctan(\frac{b}{a})<\theta\leq 2\pi-\arctan(\frac{b}{a})\end{cases}, \tag{40}$$

resulting in the constraint $ab=\pi$. For our example, we chose $a=\sqrt{0.6\pi}$ and $b=\sqrt{\frac{\pi}{0.6}}$ for an aspect ratio of 0.6.

For a star-shaped contact region:

$$T_{star}(\theta)=T_0+T_1\cos(5\theta), \tag{41}$$

resulting in the constraint $2T_0^2+T_1^2=2$. For our example, we chose $T_0=0.98$ and $T_1=0.2814$.

Using these shapes directly results in sharp corners and unrealistic artifacts, so we applied additional smoothing steps without altering the cell volume. We first constructed an additional shape with an elliptical contact region; $T(\theta)$ for an ellipse with axes c, d is:

$$T_{ellipse}(\theta)=\frac{cd}{\sqrt{(d\cos(\theta))^2+(c\sin(\theta))^2}}, \tag{42}$$

and the associated constraint is $cd=1$. We choose the aspect ratio of the ellipse to be $\frac{\text{range}(x_{contact})}{\text{range}(y_{contact})}$, where $x_{contact}$ and $y_{contact}$ are the set of coordinates within the star or rectangular contact region. Accordingly, this shape is a smoother option with a contact region that very roughly approximates the desired contour. To smooth out the cell shape away from the substrate, we can define new T functions that also depend on z:

$$T^2_{rect,smooth}(\theta,z)=\frac{z-z_{max}}{z_{max}}T^2_{rect}+\frac{z}{z_{max}}T^2_{ellipse}, \tag{43}$$

$$T^2_{star,smooth}(\theta,z)=\frac{z-z_{max}}{z_{max}}T^2_{star}+\frac{z}{z_{max}}T^2_{ellipse}, \tag{44}$$

where z_{max} is the height of the cell contour. Using these definitions, the desired contour shape at the surface is perfectly preserved and cell volume is conserved. In the case of the rectangular mesh, we also rounded off the sharp corners of the contact region, replacing them with arcs of radius 0.2 μm. The nuclear shape remained unaltered across all simulations.

Supplementary Note 5: Numerical testing and performance testing

5.1 Numerical testing of protein phosphorylation model

As an idealized test case for SMART, we considered the simple system described by Meyers et al., in which a single protein is phosphorylated at the plasma membrane with rate k_{kin} and dephosphorylated through the cytosol with rate k_p[11]. The SMART specifications are summarized in Supplementary Tables 2 to 5.

Supplementary Table 2: Compartments in phosphorylation model. Mesh statistics are given for the coarsest mesh ($h = 3.0$)

	Dimensionality	Species	Vertices	Cells	Marker value	Size
PM	2	0	242	400	10	8.000×10^2 µm^2
Cyto	3	1	363	1200	1	8.000×10^2 µm^3

Supplementary Table 3: Species in phosphorylation model.

	Compartment	D(µm^2 s^{-1})	Initial condition
Aphos	Cyto	10	1.000×10^{-1} µM

Supplementary Table 4: Reactions in phosphorylation model.

	Reactants	Products	Equation	Type
r1	[]	[‘Aphos’]	$(VolSA)k_{kin}(A_{tot} - A_{phos})$	volume_surface
r2	[‘Aphos’]	[]	$k_{dephos}A_{phos}$	volume

Supplementary Table 5: Parameters in phosphorylation model.

	Value/Equation	Description
A_tot	1.000 µM	Total cytosolic A
k_kin	5.000×10^1 s^{-1}	Rate constant for A phosphorylation
VolSA	5.000×10^{-2} µm	Cytosolic volume to PM surface area ratio
k_p	1.000×10^1 s^{-1}	Rate constant for A dephosphorylation

Given the two reactions summarized above, we can define the following flux terms in accordance with Section 1.4:

$$R^{cyto\text{-}PM}_{A_{phos},r1} = \alpha^{cyto\text{-}PM}_{A_{phos},r1}[VolSA]k_{kin}(A_{tot} - u_{A_{phos}}), \tag{45}$$

$$f^{cyto}_{A_{phos},r2} = -k_p A_{phos}. \tag{46}$$

Accordingly the governing equation and boundary conditions are:

$$\frac{\partial u_{A_{phos}}}{\partial t} = D_{A_{phos}} \nabla^2 u_{A_{phos}} + f^{cyto}_{A_{phos},r2} \quad \text{in } \Omega_{Cyto}, \tag{47}$$

$$D_{A_{phos}}(\mathbf{n} \cdot \nabla u_{A_{phos}}) = R^{cyto\text{-}PM}_{A_{phos},r1} \quad \text{on } \Gamma_{PM}. \tag{48}$$

Due to the simplicity of this problem, a closed form solution exists for the steady state concentration in 1D. This 1D solution approximates the 3D case in which a cell is modeled as a very thin sheet, where the solution is used along the thin cell dimension in the z direction. A cell of thickness Δz with one region of membrane at $z = 0$ and the other at $z = \Delta z$ is then predicted to have the following concentration profile at steady state:

$$u_{A_{phos},SS} = A_{tot}C_1\left(\exp\left(\frac{\Delta z - z}{z_0}\right) + \exp\left(\frac{z}{z_0}\right)\right), \tag{49}$$

$$\text{where} \quad C_1 = \frac{k_{phos}z_0}{D_{A_{phos}}\left(\exp\left(\frac{\Delta z}{z_0}\right) - 1\right) + k_{phos}z_0\left(1 + \exp\left(\frac{\Delta z}{z_0}\right)\right)} \tag{50}$$

$$\text{and} \quad z_0 = \sqrt{\frac{D_{A_{phos}}}{k_{dephos}}}. \tag{51}$$

We ran simulations for a thin slab with thickness ten times smaller than its length along the x and y axes, testing the effects of time-step refinement, mesh refinement, and diffusion coefficient. As an error metric, we computed the $\mathcal{L}_2$ norm of the difference between the analytical steady state solution and numerical solution at $t = 1$ s ($u_{A_{phos},num}$):

$$\left|\left|u_{A_{phos},SS} - u_{A_{phos},num}\right|\right|_{\mathcal{L}2} = \sqrt{\iiint_{\Omega_{Cyto}} (u_{A_{phos},SS} - u_{A_{phos},num})^2 d\Omega}. \tag{52}$$

Supplementary Table 6: $\mathcal{L}_2$ error (in µM) between computed and analytical solution for different time steps and mesh sizes for $D = 10.0$ µm^2 s^{-1}.

	$h = 0.375$ µm	$h = 0.75$ µm	$h = 1.5$ µm	$h = 3$ µm
$\tau = 0.64$ s	8.87×10^{-2}	8.58×10^{-2}	1.06×10^{-1}	3.68×10^{-1}
$\tau = 0.32$ s	1.97×10^{-2}	2.56×10^{-2}	8.77×10^{-2}	3.96×10^{-1}
$\tau = 0.16$ s	5.58×10^{-3}	2.24×10^{-2}	9.14×10^{-2}	4.04×10^{-1}
$\tau = 0.08$ s	5.69×10^{-3}	2.31×10^{-2}	9.21×10^{-2}	4.06×10^{-1}
$\tau = 0.04$ s	5.78×10^{-3}	2.32×10^{-2}	9.22×10^{-2}	4.06×10^{-1}
$\tau = 0.02$ s	5.80×10^{-3}	2.32×10^{-2}	9.23×10^{-2}	4.06×10^{-1}
$\tau = 0.01$ s	5.80×10^{-3}	2.32×10^{-2}	9.23×10^{-2}	4.06×10^{-1}

Supplementary Table 7: $\mathcal{L}_2$ error (in µM) between computed and analytical solution for different time steps and mesh sizes for $D = 100.0$ µm^2 s^{-1}.

	$h = 0.375$ µm	$h = 0.75$ µm	$h = 1.5$ µm	$h = 3$ µm
$\tau = 0.64$ s	9.64×10^{-2}	9.58×10^{-2}	9.35×10^{-2}	8.41×10^{-2}
$\tau = 0.32$ s	2.09×10^{-2}	2.04×10^{-2}	1.98×10^{-2}	4.27×10^{-2}
$\tau = 0.16$ s	2.53×10^{-3}	2.93×10^{-3}	9.33×10^{-3}	4.78×10^{-2}
$\tau = 0.08$ s	5.89×10^{-4}	2.40×10^{-3}	9.86×10^{-3}	4.89×10^{-2}
$\tau = 0.04$ s	6.06×10^{-4}	2.48×10^{-3}	9.96×10^{-3}	4.90×10^{-2}
$\tau = 0.02$ s	6.19×10^{-4}	2.49×10^{-3}	9.97×10^{-3}	4.90×10^{-2}
$\tau = 0.01$ s	6.22×10^{-4}	2.49×10^{-3}	9.98×10^{-3}	4.90×10^{-2}

Supplementary Table 8: $\mathcal{L}_2$ error (in µM) between computed and analytical solution for different time steps and mesh sizes for $D = 1000.0$ µm^2 s^{-1}.

	$h = 0.375$ µm	$h = 0.75$ µm	$h = 1.5$ µm	$h = 3$ µm
$\tau = 0.64$ s	9.72×10^{-2}	9.71×10^{-2}	9.69×10^{-2}	9.49×10^{-2}
$\tau = 0.32$ s	2.11×10^{-2}	2.10×10^{-2}	2.08×10^{-2}	1.92×10^{-2}
$\tau = 0.16$ s	2.65×10^{-3}	2.59×10^{-3}	2.48×10^{-3}	4.40×10^{-3}
$\tau = 0.08$ s	3.04×10^{-4}	3.27×10^{-4}	9.39×10^{-4}	4.90×10^{-3}
$\tau = 0.04$ s	6.54×10^{-5}	2.38×10^{-4}	9.91×10^{-4}	5.02×10^{-3}
$\tau = 0.02$ s	5.95×10^{-5}	2.48×10^{-4}	1.00×10^{-3}	5.04×10^{-3}
$\tau = 0.01$ s	6.12×10^{-5}	2.50×10^{-4}	1.01×10^{-3}	5.04×10^{-3}

5.2 Extra details on the numerical testing of biological test cases

In our numerical testing of the mechanotransduction and dendritic spine examples, we considered refined versions of the reference mesh. For the model of mechanotransduction, we tested refined versions of the 2D mesh used to model an axisymmetric spread cell on a circular contact region. The mesh statistics and total degrees of freedom (DOFs) are given in Supplementary Table 9.

Supplementary Table 9: Refined meshes for numerical testing of the mechanotransduction model.

Refinements	Vertices	Triangles	DOFs
0	308	548	3078
1	1163	2192	11058
2	4517	8768	41772
3	17801	35072	162216

In the case of the dendritic spine example, we tested a range of different mesh refinements and time steps, as summarized in Supplementary Figure 1. Overall, we found the effects of time-step refinement were almost identical across three different mesh refinements. We observe almost identical solutions for time steps 0.001 s or less, justifying our choice of 0.001 s time steps for the results shown in Figure 4 of the main text. At the smallest time step tested, the error due to mesh refinement is very small, as indicated by the minimal differences across mesh refinements. As shown in Figure 6E in the main text, mesh refinement does influence the spatial solution, but this effect appears to be quite small at the starting resolution of the dendritic spine mesh.

The mesh statistics for each refinement are provided in Supplementary Table 10.

Supplementary Table 10: Refined meshes for numerical testing of the dendritic spine model.

Refinements	Vertices	Tetrahedra	DOFs
0	18649	106979	49194
1	146024	855832	323328
2	1154871	6846656	2299090

5.3 Computational performance and scalability

To study the scalability of our computational framework, we consider the simulation of intracellular Ca^{2+} dynamics in an electron micrograph-based representation of a dendritic spine. Starting with the baseline computational mesh, we consider two additional levels of uniform mesh refinement resulting in three discrete representations ('standard', 'fine', and 'extra fine'). These meshes are composed of 106 979 (18 649), 855 832 (146 024), and 6 846 656 (1 154 871) tetrahedral cells (vertices), respectively, thus corresponding to an 8× increase in the number of cells for each refinement and a similar increase ($7.8 - 7.9\times$) in the number of vertices. The corresponding numbers of degrees of freedom (N, dimensions of the discrete solutions) are: 49 194, 323 328, 2 299 090; thus corresponding to a 6.6× and 7.1× increase between refinements. For each simulation, we use a 0.001 s timestep through $t = 0.025$ s. We first note that the number of nonlinear iterations per time step is constant (4) across time steps and refinement levels. Next, inspecting the total run time of each simulation as a function of the computational cost (measured in terms of the number of degrees of freedom N), we observe that the simulation time scales close to log-linearly (between $\mathcal{O}(N)$ and $\mathcal{O}(N \log N)$) (Supplementary Figure 2A). The computational cost associated with the initialization of the SMART symbolic problem representation is small (2.0%, 2.4%, 3.6%). A break-down of the total run times shows that the simulation time is persistently dominated by the finite element assembly (87.4%, 88.4%, 87.0%), followed by the iterative solution of the linear systems (9.5%, 8.4%, 8.8%) (Supplementary Figure 2B).

Model specifications for all examples and biological test cases

Here, we summarize each model in terms of its compartments, species, reactions and parameters. We note that these tables were directly output from SMART using the `print_to_latex` functions associated with each class of container, with some minor modifications for readability.

Mechanotransduction model

Supplementary Table 11: Compartments in mechanotransduction model.

	Dimensionality	Species	Vertices	Cells	Marker value	Size
Circular contact region						($\int 2\pi r dr$)
Cyto	2	11	7920	15360	1	1.925×10^3 µm^2
PM	1	2	305	304	10	1.295×10^3 µm
Nuc	2	1	649	1159	2	2.823×10^2 µm^2
NM	1	2	125	124	12	2.345×10^2 µm
Rectangular contact region						(1/4 symmetry)
Cyto	3	11	17479	84954	1	4.794×10^2 µm^3
PM	2	2	5113	10030	10	3.314×10^2 µm^2
Nuc	3	1	1668	6406	2	7.044×10^1 µm^3
NM	2	2	901	1716	12	5.856×10^1 µm^2
Star-shaped contact region						(1/10 symmetry)
Cyto	3	11	7687	34466	1	1.899×10^2 µm^3
PM	2	2	2158	4110	10	1.360×10^2 µm^2
Nuc	3	1	799	2759	2	2.818×10^1 µm^3
NM	2	2	380	674	12	2.342×10^1 µm^2

Supplementary Table 12: Species in mechanotransduction model.

	Compartment	D (µm^2 s^{-1})	Initial Condition
Emod*	PM	0	$70\left(\frac{1-\mathrm{sign}(z-0.0001)}{2}\right)$ GPa
pFAK	Cyto	10	3.000×10^{-1} µM
RhoA_GDP	Cyto	1	1.000 µM
RhoA_GTP	PM	0.3	3.360×10^1 µm^{-2}
ROCK_A	Cyto	75	0.000 µM
mDia_A	Cyto	1	0.000 µM
Myo_A	Cyto	0.8	1.500 µM
LIMK_A	Cyto	10	1.000×10^{-1} µM
Cofilin_NP	Cyto	10	1.800 µM
FActin	Cyto	0.6	1.790×10^1 µM
GActin	Cyto	13.37	4.824×10^2 µM
LaminA	NM	0.001	0.000 µm^{-2}
NPC_A	NM	0.001	0.000 µm^{-2}
YAPTAZ	Cyto	19	7.000×10^{-1} µM
YAPTAZ_phos	Cyto	19	2.000×10^{-1} µM
YAPTAZ_nuc	Nuc	19	7.000×10^{-1} µM

*Emod represents the substrate stiffness and also specifies the location of the substrate ($z = 0$).

Supplementary Table 13: Reactions in mechanotransduction model.

	Reactants	Products	Equation	Type
a1	[]	[‘pFAK’]	$(cyto_{Convert})([FAK_{tot}] - [pFAK])(\frac{E_{mod}k_{sf}}{C + E_{mod}} + k_f)$	vol-surf
a2	[‘pFAK’]	[]	$k_{df}[pFAK]$	vol
a3	[‘RhoA_GDP’]	[‘RhoA_GTP’]	$[RhoA_{GDP}]cyto_{Convert}k_{fkrho}(\gamma_{const}[pFAK]^5 + 1)$ $-[RhoA_{GTP}]k_{drho}$	vol-surf
a4	[]	[‘ROCK_A’]	$[RhoA_{GTP}]k_{rrho}([ROCK_{tot}] - [ROCK_A])$	vol-surf
a5	[‘ROCK_A’]	[]	$[ROCK_A]k_{drock}$	vol
b1	[]	[‘mDia_A’]	$[RhoA_{GTP}]k_{mrho}([mDia_{tot}] - [mDia_A])$	vol-surf
b2	[‘mDia_A’]	[]	$k_{dmdia}[mDia_A]$	vol
b3	[]	[‘Myo_A’]	$k_{mr}([Myo_{tot}] - [Myo_A])\cdot \left([ROCK_A]\epsilon\frac{1 + \tanh(sc1([ROCK_A] - [ROCK_B]))}{2} + 1\right)$ $-[Myo_A]k_{dmy}$	vol
b4	[]	[‘LIMK_A’]	$k_{lr}([LIMK_{tot}] - [LIMK_A])\cdot \left([ROCK_A]\tau\frac{1 + \tanh(sc1([ROCK_A] - [ROCK_B]))}{2} + 1\right)$ $-[LIMK_A]k_{dl}$	vol
b5	[]	[‘Cofilin_NP’]	$k_{turnover}([Cofilin_{tot}] - [Cofilin_{NP}])$ $-\frac{[Cofilin_{NP}][LIMK_A]k_{catCof}}{Cofilin_{NP} + k_{mCof}}$	vol
b6	[‘GActin’]	[‘FActin’]	$[GActin]k_{ra}\cdot \left(\alpha[mDia_A]\frac{1 + \tanh(sc1([mDia_A] - [mDia_B]))}{2} + 1\right)$ $-[FActin]([Cofilin_{NP}]k_{fc1} + k_{dep})$	vol
c1	[‘YAPTAZ_phos’]	[‘YAPTAZ’]	$[YAPTAZ_{phos}]([FActin][Myo_A]k_{CY} + k_{CN})$ $-[YAPTAZ]k_{NC}$	vol
c3	[]	[‘LaminA’]	$\frac{[FActin]^{2.6}k_{fl}p([LaminA_{tot}] - [LaminA])}{C_{LaminA} + [FActin]^{2.6}p} - [LaminA]k_{rl}$	vol-surf
c4	[]	[‘NPC_A’]	$[FActin][LaminA][Myo_A]k_{fNPC}([NPC_{tot}] - [NPC_A])$ $-[NPC_A]k_{rNPC}$	vol-surf

Continued on next page

	Reactants	Products	Equation	Type
c5	[‘YAPTAZ’]	[‘YAPTAZ_nuc’]	$[YAPTAZ]([NPC_A]k_{in2} + k_{insolo}) - [YAPTAZ_{nuc}]k_{out}$	vol-surf-vol

Supplementary Table 14: Parameters in mechanotransduction model. Values were adopted directly from those reported in Ref. 9

	Value	Description
FAK_tot	1.000 µM	Total cytosolic FAK
k_f	1.500×10^{-2} s^{-1}	rate constant for baseline FAK phosphorylation
k_sf	3.790×10^{-1} s^{-1}	substrate-stiffness-dependent FAK phosphorylation rate constant
C	3.250 kPa	critical substrate stiffness
cytoConvert	1.825 µm	vol/SA ratio
k_df	3.500×10^{-2} s^{-1}	FAK dephosphorylation rate constant
k_fkrho	1.680×10^{-2} s^{-1}	RhoA activation rate constant
gammaConst	7.756×10^{1} µM^{-5}	Scaling factor
k_drho	6.250×10^{-1} s^{-1}	RhoA deactivation rate constant
k_rrho	6.480×10^{-1} µM^{-1} s^{-1}	ROCK activation rate constant
ROCK_tot	1.000 µM	Total cytosolic ROCK
k_drock	8.000×10^{-1} s^{-1}	ROCK deactivation rate constant
k_mrho	2.000×10^{-3} µM^{-1} s^{-1}	mDia activation rate constant
mDia_tot	8.000×10^{-1} µM	Total cytosolic mDia
k_dmdia	5.000×10^{-3} s^{-1}	mDia deactivation rate constant
Myo_tot	5.000 µM	Total cytosolic myosin
k_mr	3.000×10^{-2} s^{-1}	Myosin activation rate constant
ROCK_B	3.000×10^{-1} µM	Critical activated ROCK concentration
epsilon	3.600×10^{1} µM^{-1}	Scaling factor
sc1	2.000×10^{1} µM^{-1}	Scaling factor
k_dmy	6.700×10^{-2} s^{-1}	Myosin deactivation rate constant
LIMK_tot	2.000 µM	Total cytosolic LIMK
k_lr	7.000×10^{-2} s^{-1}	LIMK activation rate constant
tau	5.549×10^{1} µM^{-1}	Scaling factor
k_dl	2.000 s^{-1}	LIMK deactivation rate constant
Cofilin_tot	2.000 µM	Total cytosolic cofilin
k_turnover	4.000×10^{-2} s^{-1}	Cofilin dephosphorylation rate constant
k_catCof	3.400×10^{-1} s^{-1}	Cofilin phosphorylation rate constant
k_mCof	4.000 µM	Critical cofilin concentration
k_ra	4.000×10^{-1} s^{-1}	Actin polymerization rate constant
alpha	5.000×10^{1} µM^{-1}	Scaling factor
mDia_B	1.650×10^{-1} µM	Critical activated mDia concentration
k_dep	3.500 s^{-1}	Actin depolymerization rate constant
k_fc1	4.000 µM^{-1} s^{-1}	Cofilin-mediated actin polymerization rate constant
k_CN	5.600×10^{-1} s^{-1}	Baseline YAP/TAZ dephosphorylation rate constant
k_CY	7.600×10^{-4} µM^{-2} s^{-1}	stress-fiber-mediated YAP/TAZ dephosphorylation rate constant
k_NC	1.400×10^{-1} s^{-1}	YAP/TAZ phosphorylation rate constant
LaminA_tot	3.500×10^{3} µm^{-2}	Total lamin A in NM
k_fl	4.600×10^{-1} s^{-1}	Lamin A dephosphorylation rate constant
p	9.000×10^{-6} kPa µM$^{-2.6}$	Scaling factor for cell stiffness
C_LaminA	1.000×10^{2} kPa	Critical cell stiffness for lamin A dephosphorylation
k_rl	1.000×10^{-3} s^{-1}	Lamin A phosphorylation rate constant
NPC_tot	6.500 µm^{-2}	Total NPC density in NM
k_fNPC	2.800×10^{-7} µm^{2} µM^{-2} s^{-1}	NPC opening rate constant
k_rNPC	8.700 s^{-1}	NPC closing rate constant

Continued on next page

	Value	Description
k_insolo	$1.000\,\mu m^{-2}\,\mu M^{-1}\,s^{-1}$	NPC-independent YAP/TAZ nuclear import rate
k_in2	$1.000 \times 10^{1}\,\mu M^{-1}\,s^{-1}$	NPC-dependent YAP/TAZ nuclear import rate
k_out	$1.000\,\mu m^{-2}\,\mu M^{-1}\,s^{-1}$	YAP/TAZ nuclear export rate

Dendritic spine calcium model

Supplementary Table 15: Compartments in dendritic spine model.

	Dimensionality	Species	Vertices	Cells	Marker value	Size
Cyto	3	2	17742	81206	1	$6.463 \times 10^{-1}\,\mu m^3$
PM	2	3	1661	3243	10	$6.236\,\mu m^2$
SA	3	1	8727	25773	2	$2.692 \times 10^{-2}\,\mu m^3$
SAm	2	0	7820	15672	12	$2.387\,\mu m^2$

Supplementary Table 16: Species in dendritic spine model.

	Compartment	D ($\mu m^2\,s^{-1}$)	Initial Condition
Ca	Cyto	220	$1.000 \times 10^{-1}\,\mu M$
NMDAR	PM	0	1.000^*
VSCC	PM	0	$\frac{1 + \mathrm{sign}(z + 0.25)}{2}$
Bf	PM	0	$7.957\,\mu m\,\mu M$
Bm	Cyto	20	$2.000 \times 10^{1}\,\mu M$
CaSA	SA	45	$6.000 \times 10^{1}\,\mu M$

*NMDAR restricted to postsynaptic density.

Supplementary Table 17: Reactions in dendritic spine model.

	Reactants	Products	Equation	Type
a1	[]	[‘Ca^{2+}’]	$G_{NMDAR}(J0_{NMDAR})[NMDAR](V_m - V_{rev})$	volume_surface
a2	[]	[‘Ca^{2+}’]	$J_{VSCC}[VSCC]$	volume_surface
a3	[‘Ca^{2+}’]	[]	$\dfrac{100n_{PMr}\left(\dfrac{[Ca^{2+}]^5(Vmax_{hr23})}{[Ca^{2+}]^5 + (Km_{hr23})^5} + \dfrac{[Ca^{2+}]^2(Vmax_{lr23})}{[Ca^{2+}]^2 + (Km_{lr23})^2}\right)}{1 + \dfrac{(Kme)(Prtote)}{([Ca^{2+}] + (Kme))^2} + \dfrac{(Kmx)(Prtotex)}{([Ca^{2+}] + (Kmx))^2}}$	volume_surface
a4	[‘Ca^{2+}’]	[]	$\dfrac{1000n_{PMr}\dfrac{(Vmax_{r22})[Ca^{2+}]}{[Ca^{2+}] + (Km_{r22})}}{1 + \dfrac{(Kme)(Prtote)}{([Ca^{2+}] + (Kme))^2} + \dfrac{(Kmx)(Prtotex)}{([Ca^{2+}] + (Kmx))^2}}$	volume_surface
a5	[‘Ca^{2+}’, ‘Bf’]	[]	$(kBf_{on})[Bf][Ca^{2+}] - (kBf_{off})((Bf_{tot}) - [Bf])$	volume_surface
b1	[‘Ca^{2+}’, ‘Bm’]	[]	$(kBm_{on})[Bm][Ca^{2+}] - (kBm_{off})((Bm_{tot}) - [Bm])$	volume

Continued on next page

	Reactants	Products	Equation	Type
c1	[‘Ca^{2+}’]	[‘CaSA’]	$\dfrac{1000 n_{SAr}\dfrac{[Ca^{2+}]^2(Vmax_{r19})}{[Ca^{2+}]^2+(KP_{r19})^2}}{1+\dfrac{(Kme)(Prtote)}{([Ca^{2+}]+(Kme))^2}+\dfrac{(Kmx)(Prtotex)}{([Ca^{2+}]+(Kmx))^2}}$	volume_surface_volume
c2	[‘CaSA’]	[‘Ca^{2+}’]	$k_{leak} n_{SAr}([CaSA]-[Ca^{2+}])$	volume_surface_volume

Supplementary Table 18: Parameters in dendritic spine model.

	Value/Equation	Description
n_PMr	1.011×10^{-1} µm	Experimental vol to surface area ratio
Vm	$\Big[\left(55.51\exp\left(-\frac{t}{0.003}\right)+10.29\exp(-40.0t)\right)\frac{\mathrm{sign}(t)+1}{2}$ $+25\left(\exp\left(-\frac{t}{0.05}\right)-\exp\left(-\frac{t}{0.005}\right)\right)\frac{\mathrm{sign}(t)+1}{2}-65\Big]$ mV	PM voltage
Vrev	9.000×10^{1} mV	Reversal voltage for NMDAR
G_NMDAR	1.377×10^{5} $\mathrm{mV^{-1}\,s^{-1}}$	NMDAR conductance
J0_NMDAR	$-\dfrac{0.00456\left(\exp\left(-\frac{t}{0.05}\right)+\exp\left(-\frac{t}{0.05}\right)\right)(\mathrm{sign}(t)+1)}{110.8\exp(-0.092V_m)+1}$ $\mu\mathrm{m}^{-2}$	Ca^{2+} influx through NMDAR
J_VSCC	$(0.393-2.245\exp(-0.01044V_m)(\exp(-34700t)-\exp(-3680t))\cdot$ $(\mathrm{sign}(t)+1)\dfrac{-1120(2V_m+1456)}{192970-85942\exp(0.01044Vm)}\mathrm{s^{-1}}\ \mu\mathrm{m}^{-2}$	Ca^{2+} influx through VSCC
Prtote	1.910×10^{2} µM	Ca^{2+} buffer 1 concentration
Kme	2.430 µM	Ca^{2+} buffer 1 affinity
Prtotex	8.770 µM	Ca^{2+} buffer 2 concentration
Kmx	1.390×10^{-1} µM	Ca^{2+} buffer 2 affinity
Vmax_lr23	1.130×10^{-1} $\mu\mathrm{M\,s^{-1}}$	PMCA conductance 1
Km_lr23	4.420×10^{-1} µM	PMCA activation constant 1
Vmax_hr23	5.900×10^{-1} $\mu\mathrm{M\,s^{-1}}$	PMCA conductance 2
Km_hr23	4.420×10^{-1} µM	PMCA activation constant 2
Vmax_r22	1.000×10^{-1} $\mu\mathrm{M\,s^{-1}}$	NCX conductance
Km_r22	1.000 µM	NCX activation constant
kBf_on	1.000 $\mu\mathrm{M^{-1}\,s^{-1}}$	On-rate for fixed Ca^{2+} buffer
kBf_off	2.000 $\mathrm{s^{-1}}$	Off-rate for fixed Ca^{2+} buffer
Bf_tot	7.957 µm µM	Total amount of fixed Ca^{2+} buffer
kBm_on	1.000 $\mu\mathrm{M^{-1}\,s^{-1}}$	On-rate for mobile Ca^{2+} buffer
kBm_off	1.000 $\mathrm{s^{-1}}$	Off-rate for mobile Ca^{2+} buffer
Bm_tot	2.000×10^{1} µM	Total conc. of mobile Ca^{2+} buffer
n_SAr	1.130×10^{-2} µm	Cytosolic volume to SA surf. area ratio
Vmax_r19	1.140×10^{2} $\mu\mathrm{M\,s^{-1}}$	SERCA conductance
KP_r19	2.000×10^{-1} µM	SERCA activation constant
k_leak	1.608×10^{-1} $\mathrm{s^{-1}}$	SR Ca^{2+} leak rate constant

Calcium release unit model

Supplementary Table 19: Compartments in CRU model.

	Dimensionality	Species	Vertices	Cells	Marker value	Size
Cyto	3	4	45557	204894	1	8.029×10^8 nm^3
SR	3	2	12178	42030	2	3.438×10^6 nm^3
TTM	2	0	13282	26552	10	7.190×10^5 nm^2
SRM	2	1	9749	19518	12	4.129×10^5 nm^2

Supplementary Table 20: Species in CRU model.

	Compartment	D (µm^2 s^{-1})	Initial Condition
Ca	Cyto	220	1.400×10^{-1} µM
ATP	Cyto	140	4.547×10^2 µM
CMDN	Cyto	25	2.353×10^1 µM
TRPN	Cyto	0	$\dfrac{56.8}{1+\exp\left(-\dfrac{(x+125)^2+(z+75)^2}{100^2}\right)}$ µM *
CaSR	SR	220	1.300×10^3 µM
CSQN	SR	25	2.110×10^3 µM
RyR	SRM	0	1.000**

* TRPN distribution chosen to approximate that in Ref. 12, using the distance from a central point in the T-tubule geometry rather than the distance to the junctional T-tubule boundary.
** As in Ref. 12, RyR was localized to the SR-T-tubule junction; here defined as any region of the SR membrane less than 20 nm from the T-tubule membrane.

Supplementary Table 21: Reactions in CRU model.

	Reactants	Products	Equation	Type
s1	[‘CaSR’]	[‘Ca^{2+}’]	$(NO_{RyR})[RyR]g_{RyR}([CaSR]-[Ca^{2+}])$	vol-surf-vol
s2*	[‘Ca^{2+}’]	[‘CaSR’]	$2(SR_{VtoA})S_{SERCA}\rho_{SERCA}\cdot \dfrac{[Ca^{2+}]^2\alpha_{1,plus}\alpha_{2,plus}-[CaSR]^2\alpha_{1,minus}\alpha_{2,minus}}{[Ca^{2+}]^2\alpha_{1,plus}+[CaSR]^2\alpha_{1,minus}+\alpha_{2,minus}+\alpha_{2,plus}}$	vol-surf-vol
t1	[]	[‘Ca^{2+}’]	$(Jmax)\left(1+(VFactor2)(ksat)\right)\cdot \dfrac{(CaTT)(Nai)^3(VFactor1)-[Ca^{2+}](Nao)^3(VFactor2)}{[Ca^{2+}](denom2)+(denom1)}$	vol-surf
t2	[‘Ca^{2+}’]	[]	$(Jp_{max})\dfrac{(Km_{pCa})^2}{[Ca^{2+}]^2+(Km_{pCa})^2}$	vol-surf
t3	[‘Ca^{2+}’]	[]	$(JCab)\left((Voltage)-0.5\dfrac{(R)(T)}{F}\log\left(\dfrac{(CaTT)}{[Ca^{2+}]}\right)\right)$	vol-surf

Continued on next page

	Reactants	Products	Equation	Type
b1	[‘Ca^{2+}’, ‘ATP’]	[]	$(kon_{ATP})[ATP][Ca^{2+}]$ $-(koff_{ATP})((ATP_{tot}) - [ATP])$	vol
b2	[‘Ca^{2+}’, ‘CMDN’]	[]	$(kon_{CMDN})[CMDN][Ca^{2+}]$ $-(koff_{CMDN})((CMDN_{tot}) - [CMDN])$	vol
b3	[‘Ca^{2+}’, ‘TRPN’]	[]	$(kon_{TRPN})[Ca^{2+}][TRPN]$ $-(koff_{TRPN})((TRPN_{tot}) - [TRPN])$	vol
b4	[‘CaSR’, ‘CSQN’]	[]	$(kon_{CSQN})[CSQN][CaSR]$ $-(koff_{CSQN})((CSQN_{tot}) - [CSQN])$	vol

*In simulations without SERCA, this reaction was excluded from the model.

Supplementary Table 22: Parameters in CRU model.

	Value	Description
ATP_tot	4.550×10^{2} µM	Total cytosolic ATP
CMDN_tot	2.400×10^{1} µM	Total cytosolic CMDN
TRPN_tot	7.000×10^{1} µM	Total cytosolic TRPN
CSQN_tot	6.390×10^{3} µM	Total cytosolic CSQN
CaTT	1.800×10^{3} µM	Ca^{2+} conc. in T-tubules
NO_RyR	5.000	Number of open RyR in release event
g_RyR	2.620×10^{4} nm s^{-1}	RyR conductance
S_SERCA	1.500	SERCA scale parameter
rho_SERCA	7.500×10^{1} µM	Volume density of SERCA
SR_VtoA	3.070×10^{2} nm	cytosolic volume to SA surf. area ratio 0
alpha1_plus	1.067×10^{2} µM^{-2} s^{-1}	SERCA rate constant
alpha2_plus	5.350 s^{-1}	SERCA rate constant
alpha1_minus	5.069×10^{-5} µM^{-2} s^{-1}	SERCA rate constant
alpha2_minus	3.868×10^{-2} s^{-1}	SERCA rate constant
Voltage	-8.200×10^{-2} V	TT voltage
R	8.310 J mol^{-1} K^{-1}	Universal gas constant
T	2.9515×10^{2} K	Temperature
F	9.650×10^{4} C mol^{-1}	Faraday’s constant
Jmax	4.083×10^{2} µm µM s^{-1}	Max. NCX flux
Nai	1.420×10^{4} µM	Cytosolic sodium conc.
Nao	1.400×10^{5} µM	Extracellular sodium conc.
VFactor1	3.233×10^{-1}	NCX lumped parameter
VFactor2	8.142	NCX lumped parameter
ksat	2.700×10^{-1}	NCX saturation parameter
denom1	3.852×10^{16} µM^{4}	NCX lumped parameter
denom2	4.278×10^{15} µM^{3}	NCX lumped parameter
Jp_max	4.974 µm µM s^{-1}	Max. Ca^{2+} pump flux
Km_pCa	2.890×10^{-1} µM	Ca^{2+} pump Ca^{2+} affinity
JCab	2.611×10^{2} µm µM s^{-1} V^{-1}	TT Ca^{2+} leak flux
kon_ATP	2.250×10^{2} µM^{-1} s^{-1}	Ca^{2+}-ATP binding on rate
koff_ATP	4.500×10^{4} s^{-1}	Ca^{2+}-ATP binding off rate

Continued on next page

	Value	Description
kon_CMDN	3.400×10^1 µM^{-1} s^{-1}	Ca^{2+}-CMDN binding on rate
koff_CMDN	2.380×10^2 s^{-1}	Ca^{2+}-CMDN binding off rate
kon_TRPN	3.270×10^1 µM^{-1} s^{-1}	Ca^{2+}-TRPN binding on rate
koff_TRPN	1.960×10^1 s^{-1}	Ca^{2+}-TRPN binding off rate
kon_CSQN	1.020×10^2 µM^{-1} s^{-1}	Ca^{2+}-CSQN binding on rate
koff_CSQN	6.500×10^4 s^{-1}	Ca^{2+}-CSQN binding off rate

ATP production in mitochondria

Supplementary Table 23: Compartments in ATP generation model.

	Dimensionality	Species	Vertices	Cells	Marker value	Size
IMS	3	1	36667	128395	1	2.031×10^{-2} µm^3
OM	2	0	11514	23024	10	6.320×10^{-1} µm^2
Mat	3	2	22974	85773	2	1.647×10^{-2} µm^3
IM	2	13	18708	37598	12	1.545 µm^2

*In simulations with cristae-localized membrane species, the IM was split into two regions as shown in Figure 5B in the main text. The cristae portion had an area of 1.0197 µm ^{2}, with 28578 total surface elements.

Supplementary Table 24: Species in ATP generation model.

	Compartment*	D (µm^2 s^{-1})	Initial Condition **
E_Mat	IM	0	0.000 µm^{-2}
E_IMS	IM	0	$1.728 \times 10^2/2.618 \times 10^2$ µm^{-2}
E_Mat_H3Star	IM	0	0.000 µm^{-2}
E_Mat_H3S	IM	0	0.000 µm^{-2}
E_Mat_H3	IM	0	0.000 µm^{-2}
L	IM	0	$1.066 \times 10^4/1.615 \times 10^4$ µm^{-2}
TL	IM	0	0.000 µm^{-2}
LT	IM	0	0.000 µm^{-2}
DL	IM	0	0.000 µm^{-2}
LD	IM	0	0.000 µm^{-2}
TLD	IM	0	0.000 µm^{-2}
DLT	IM	0	0.000 µm^{-2}
DLD †	IM	0	0.000 µm^{-2}
D_Mat	Mat	15	7.200×10^{-1} mM
T_Mat	Mat	15	6.500×10^{-1} mM
T_IMS	IMS	15	3.250×10^{-1} mM

*In simulations with cristae-localized membrane species, all species listed as "IM" here were localized to the Cristae compartment instead.
** Two values are given for some species - first value associated with uniform distribution of proteins in the IM, second value for cristae-localized proteins, ensuring consistency in total number of molecules.
† Note that TLT' and DLD' introduced in Ref. 13 are not used here, as they are indistinguishable states that can be eliminated by substituting $TLT \leftarrow TLT + TLT'$ and $DLD \leftarrow DLD + DLD'$.

Supplementary Table 25: Reactions in ATP generation model.

	Reactants	Products	Equation	Type
E1	[‘E_Mat’]	[‘E_IMS’]	$k_{16}E_{Mat} - k_{61}E_{IMS}$	surf
E2	[‘E_IMS’]	[]	$E_{IMS}k_{65} - k_{56}E_{IMS,H3}$ *	surf
E3	[]	[‘E_Mat_H3Star’]	$k_{54}E_{IMS,H3} - E_{Mat,H3Star}k_{45}$ *	surf
E4	[]	[‘E_Mat_H3’]	$k_{52}E_{IMS,H3} - E_{Mat,H3}k_{25}$ *	surf
E5	[‘E_Mat_H3Star’, ‘D_Mat’]	[‘E_Mat_H3S’]	$k_{43}E_{Mat,H3Star}D_{Mat} - k_{34}E_{Mat,H3S}$	vol-surf
E6	[‘E_Mat_H3S’]	[‘E_Mat_H3’, ‘T_Mat’]	$k_{32}E_{Mat_{H}3S} - k_{23}E_{Mat,H3}T_{Mat}$	vol-surf
E7	[‘E_Mat_H3’]	[‘E_Mat’]	$k_{21}E_{Mat,H3} - k_{12}E_{Mat}$	surf
L1	[‘L’, ‘T_Mat’]	[‘LT’]	$k_{on,Tm}[L]T_{Mat} - k_{off,Tm}[LT]$	vol-surf
L2	[‘L’, ‘D_Mat’]	[‘LD’]	$k_{on,Dm}[L]D_{Mat} - k_{off,Dm}[LD]$	vol-surf
L3	[‘L’, ‘T_IMS’]	[‘TL’]	$k_{on,Ti}[L]T_{IMS} - k_{off,Ti}[TL]$	vol-surf
L4	[‘L’]	[‘DL’]	$D_{IMS}[L]k_{on,Di} - [DL]k_{off,Di}$	surf
L5	[‘TL’, ‘T_Mat’]	[]	$[TL]T_{Mat}k_{on,Tm} - k_{off,Tm}[TLT]$ **	vol-surf
L6	[‘TL’, ‘D_Mat’]	[‘TLD’]	$k_{on,Dm}[TL]D_{Mat} - k_{off,Dm}[TLD]$	vol-surf
L7	[‘LT’, ‘T_IMS’]	[]	$[LT]T_{IMS}k_{on,Ti} - k_{off,Ti}[TLT]$ **	vol-surf
L8	[‘LT’]	[‘DLT’]	$D_{IMS}[LT]k_{on,Di} - [DLT]k_{off,Di}$	surf
L9	[‘DL’, ‘T_Mat’]	[‘DLT’]	$k_{on,Tm}[DL]T_{Mat} - k_{off,Tm}[DLT]$	vol-surf
L10	[‘DL’, ‘D_Mat’]	[‘DLD’]	$k_{on,Dm}[DL]D_{Mat} - k_{off,Dm}[DLD]$	vol-surf
L11	[‘LD’, ‘T_IMS’]	[‘TLD’]	$k_{on,Ti}[LD]T_{IMS} - k_{off,Ti}[TLD]$	vol-surf
L12	[‘LD’]	[‘DLD’]	$D_{IMS}[LD]k_{on,Di} - [DLD]k_{off,Di}$	surf
L13	[‘DLT’]	[‘TLD’]	$k_p[DLT] - k_{cp}[TLD]$	surf
V1	[‘T_IMS’]	[‘T_cyto’]	$[VDAC]k_{vdac}(T_{IMS} - T_{cyto})$	vol-surf

* Due to mass conservation, $E_{IMS,H3}$ is not treated as a separate variable, but solved for as $E_{IMS,H3} = E_{tot} - E_{Mat} - E_{IMS} - E_{Mat,H3Star} - E_{Mat,H3} - E_{Mat,H3S}$.
** Due to mass conservation, TLT is not treated as a separate variable, but solved for as $TLT = L_{tot} - L - LT - TL - LD - DL - DLD - DLT - TLD$

Supplementary Table 26: Parameters for mitochondrial ATP generation.

	Value	Description
E_tot	1.73×10^{2} µm^{-2}	Total ATP synthase
L_tot	1.07×10^{4} µm^{-2}	Total ANTs
D_IMS	4.50×10^{-2} mM	IMS ADP concentration
k_16	1.48×10^{5} s^{-1}	ATP-synthase-associated rate constants
k_61	3.37×10^{4} s^{-1}	
k_65	3.97×10^{3} s^{-1}	
k_56	1.00×10^{3} s^{-1}	
k_54	1.00×10^{2} s^{-1}	
k_45	1.00×10^{2} s^{-1}	
k_52	1.00×10^{-20} s^{-1}	
k_25	5.85×10^{-30} s^{-1}	
k_43	2.00×10^{3} mM^{-1} s^{-1}	
k_34	1.00×10^{2} s^{-1}	

Continued on next page

	Value	Description
k_32	$5.00 \times 10^{3}\,s^{-1}$	
k_23	$5.00 \times 10^{3}\,mM^{-1}\,s^{-1}$	
k_21	$4.00 \times 10^{1}\,s^{-1}$	
k_12	$1.00 \times 10^{2}\,s^{-1}$	
koff_Tm	$4.00 \times 10^{4}\,s^{-1}$	ANT-associated rate constants
kon_Tm	$6.40 \times 10^{3}\,mM^{-1}\,s^{-1}$	
koff_Ti	$2.00 \times 10^{2}\,s^{-1}$	
kon_Ti	$4.00 \times 10^{2}\,mM^{-1}\,s^{-1}$	
koff_Dm	$4.00 \times 10^{4}\,s^{-1}$	
kon_Dm	$4.00 \times 10^{3}\,mM^{-1}\,s^{-1}$	
koff_Di	$1.00 \times 10^{2}\,s^{-1}$	
kon_Di	$4.00 \times 10^{3}\,mM^{-1}\,s^{-1}$	
k_p	$9.20 \times 10^{1}\,s^{-1}$	
k_cp	$3.50\,s^{-1}$	
k_vdac	$1.00 \times 10^{3}\,mM^{-1}\,s^{-1}$	opening rate of VDACs
VDAC	$1.00 \times 10^{4}\,\mu m^{-2}$	Density of VDACs in OM

Supplementary Figures

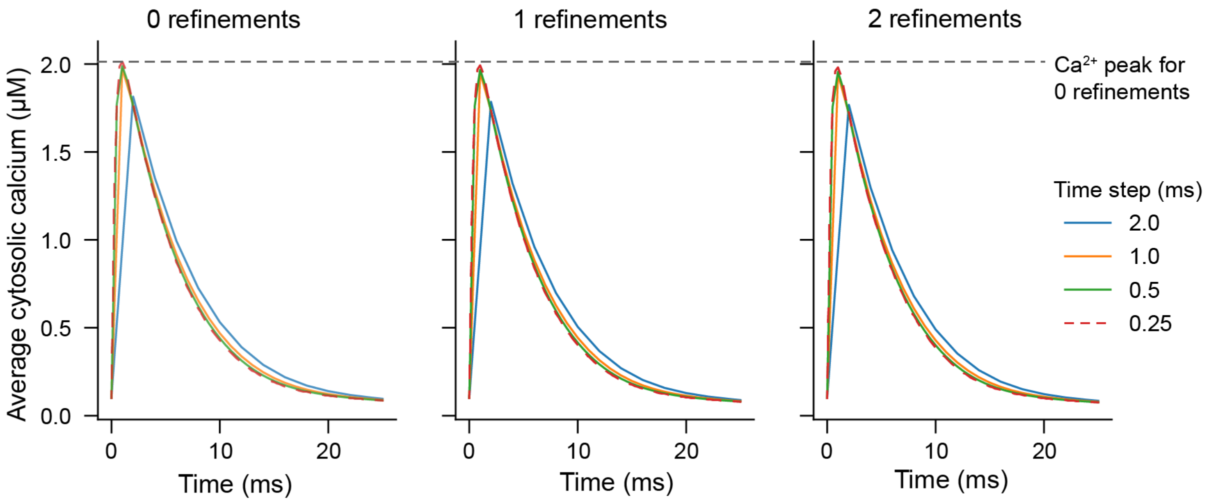

Supplementary Figure 1: Summary of calcium dynamics in dendritic spine upon mesh refinement and time-step refinement. Curves are plotted for three different mesh refinements and 5 different time steps, as indicated. The Ca^{2+} peak for the coarsest mesh is indicated by the horizontal dashed line throughout, showing the reduction in Ca^{2+} peak upon mesh refinement.

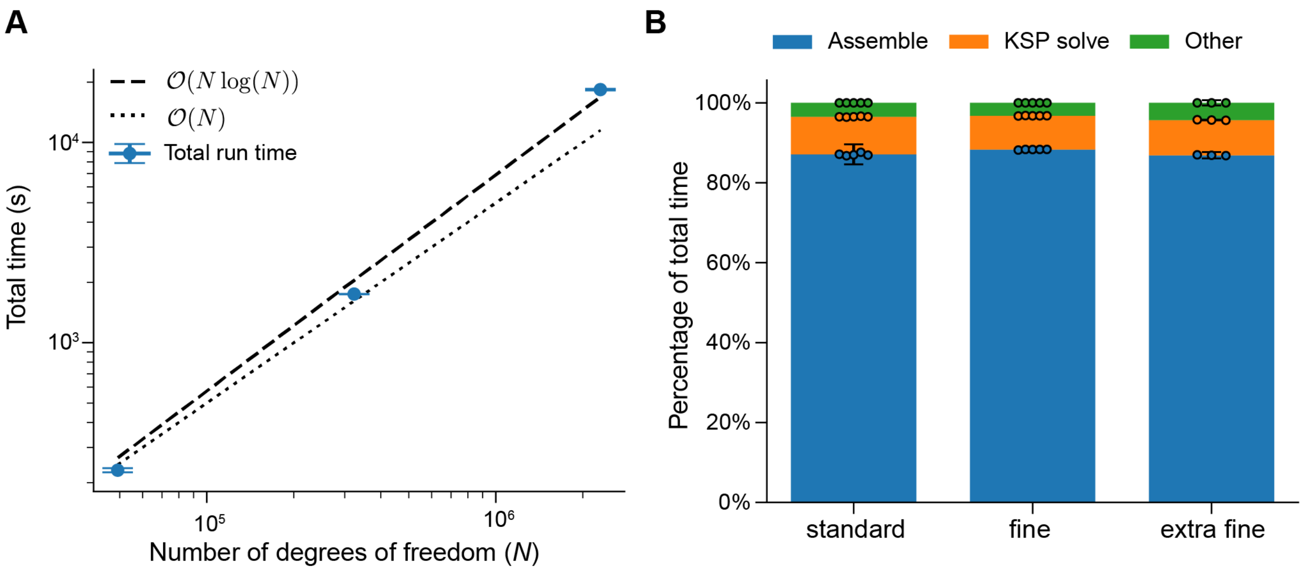

Supplementary Figure 2: Performance and scalability of SMART for dendritic spine Ca^{2+} dynamics A) Total run times for 25 simulated time steps of intracellular Ca^{2+} dynamics for increasing mesh refinement levels (standard, fine and extra fine), averaged over 5 runs for standard and fine meshes and 3 runs for the extra fine mesh. B) Relative contributions to the total run time for each level, averaged over 5 runs for standard and fine meshes and 3 runs for the extra fine mesh. The computational cost is dominated by the finite element assembly and function evaluation due to the complexity of the coupled nonlinear equations. In both A and B, data are presented as mean values ± standard deviation.

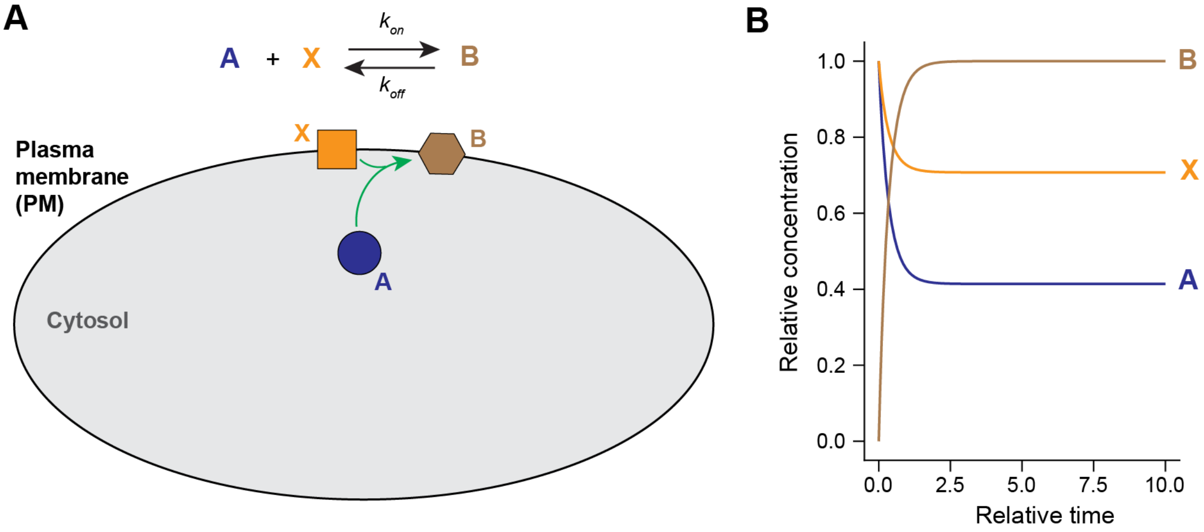

Supplementary Figure 3: Simple example of a bulk-surface reaction in a cell. A) Schematic summarizing the reaction of volume species A with surface species X to form surface species B. B) Normalized concentration over time for each of the three species.

Supplementary Videos

Each video below was created using Paraview[14].

Supplementary Video 1: F-actin and YAP/TAZ dynamics in a cell on a circular micropattern. F-actin concentration in the cytosol and YAP/TAZ concentration in the nucleus plotted within a cell on a circular contact region with a radius of 13 μm. Substrate stiffness matches that of a glass coverslip (70 GPa).

Supplementary Video 2: F-actin and YAP/TAZ dynamics in a cell on a rectangular micropattern. F-actin concentration in the cytosol and YAP/TAZ concentration in the nucleus plotted within a cell on a rectangular contact region with the same contact area and cell volume as the cell in Supplementary Video 1. Substrate stiffness matches that of a glass coverslip (70 GPa).

Supplementary Video 3: F-actin and YAP/TAZ dynamics in a cell on a star-shaped micropattern. F-actin concentration in the cytosol and YAP/TAZ concentration in the nucleus plotted within a cell on a star-shaped contact region with the same contact area and cell volume as the cell in Supplementary Video 1. Substrate stiffness matches that of a glass coverslip (70 GPa).

Supplementary Video 4: Calcium dynamics in a dendritic spine. Ca^{2+} influx into the dendritic spine over the duration of a single voltage peak. Both cytosolic and SA Ca^{2+} concentrations are shown, with the SA in the spine head accumulating Ca^{2+} at later time points due to entry via SERCA.

Supplementary Video 5: Calcium dynamics in a cardiomyocyte calcium release unit (CRU) with SERCA. Cytosolic and SR Ca^{2+} concentrations during a single Ca^{2+} release event from the SR. Ca^{2+} is slowly replenished in the SR after release due to entry through SERCA. A smaller Ca^{2+} flux occurs through the T-tubules (dark grey) and the two mitochondria (light grey) act as diffusional barriers.

Supplementary Video 6: Calcium dynamics in a cardiomyocyte calcium release unit (CRU) without SERCA. Cytosolic and SR Ca^{2+} concentrations during a single Ca^{2+} release event from the SR. In contrast to Supplementary Video 5, Ca^{2+} remains depleted in the SR here as SERCA is not present in the SR membrane.

Supplementary Video 7: ATP dynamics in a mitochondrion. ATP concentrations in the matrix (innermost volume) and intermembrane space (IMS; region between the outer and inner membranes) are displayed over 100 ms of simulation. Rapid initial decrease in both compartments is followed by a slower increase of IMS ATP and a slow decrease in matrix ATP.

References

[1] Hucka, M. *et al.* The systems biology markup language (SBML): A medium forrepresentation and exchange of biochemical network models. *Bioinformatics* **19**, 524–531, DOI: 10.1093/bioinformatics/btg015 (2003).

[2] Rangamani, P. *et al.* Decoding Information in Cell Shape. *Cell* **154**, 1356–1369, DOI: 10.1016/j.cell.2013.08.026 (2013).

[3] Alnæs, M. *et al.* The FEniCS Project Version 1.5. *<p>Archive of Numerical Software* **Vol 3**, Starting Point and Frequency: Year: 2013</p>, DOI: 10.11588/ANS.2015.100.20553 (2015).

[4] Alnæs, M. S., Logg, A., Ølgaard, K. B., Rognes, M. E. & Wells, G. N. Unified form language: A domain-specific language for weak formulations of partial differential equations. *ACM Transactions on Mathematical Software* **40**, 9:1–9:37, DOI: 10.1145/2566630 (2014).

[5] Balay, S., Gropp, W., McInnes, L. C. & Smith, B. F. PETSc, the portable, extensible toolkit for scientific computation (1998).

[6] Grecco, H. E. Pint: Physical quantities module.

[7] Dalcin, L. D., Paz, R. R., Kler, P. A. & Cosimo, A. Parallel distributed computing using Python. *Advances in Water Resources* **34**, 1124–1139, DOI: 10.1016/j.advwatres.2011.04.013 (2011).

[8] Geuzaine, C. & Remacle, J.-F. Gmsh: A 3-D finite element mesh generator with built-in pre- and post-processing facilities: THE GMSH PAPER. *International Journal for Numerical Methods in Engineering* **79**, 1309–1331, DOI: 10.1002/nme.2579 (2009).

[9] Scott, K. E., Fraley, S. I. & Rangamani, P. A spatial model of YAP/TAZ signaling reveals how stiffness, dimensionality, and shape contribute to emergent outcomes. *Proceedings of the National Academy of Sciences* **118**, e2021571118, DOI: 10.1073/pnas.2021571118 (2021).

[10] Meurer, A. *et al.* SymPy: Symbolic computing in Python. *PeerJ Computer Science* **3**, e103, DOI: 10.7717/peerj-cs.103 (2017).

[11] Meyers, J., Craig, J. & Odde, D. J. Potential for Control of Signaling Pathways via Cell Size and Shape. *Current Biology* **16**, 1685–1693, DOI: 10.1016/j.cub.2006.07.056 (2006).

[12] Hake, J. *et al.* Modelling cardiac calcium sparks in a three-dimensional reconstruction of a calcium release unit: Calcium sparks in reconstructed release unit. *The Journal of Physiology* **590**, 4403–4422, DOI: 10.1113/jphysiol.2012.227926 (2012).

[13] Garcia, G. C., Gupta, K., Bartol, T. M., Sejnowski, T. J. & Rangamani, P. Mitochondrial morphology governs ATP production rate. *Journal of General Physiology* **155**, DOI: 10.1085/jgp.202213263 (2023).

[14] Ahrens, J., Geveci, B. & Law, C. ParaView: An End-User Tool for Large-Data Visualization. In *Visualization Handbook*, 717–731, DOI: 10.1016/B978-012387582-2/50038-1 (Elsevier, 2005).
