# Supplementary material for: Spatial modeling algorithms for reactions and transport in biological cells
Source: Nat Comput Sci. 2024 Dec 19;5(1):76–89. doi: 10.1038/s43588-024-00745-x (PMC11774757; doi:10.1038/s43588-024-00745-x)
Supplement: Supplementary file 2 — Reporting Summary [file 43588_2024_745_MOESM2_ESM.pdf]

nature portfolio

Corresponding author(s): Padmini Rangamani and Marie E. Rognes

Last updated by author(s): Nov 6, 2024

Reporting Summary

Nature Portfolio wishes to improve the reproducibility of the work that we publish. This form provides structure for consistency and transparency in reporting. For further information on Nature Portfolio policies, see our Editorial Policies and the Editorial Policy Checklist.

Statistics

For all statistical analyses, confirm that the following items are present in the figure legend, table legend, main text, or Methods section.

n/a	Confirmed	
☐	☒	The exact sample size (*n*) for each experimental group/condition, given as a discrete number and unit of measurement
☒	☐	A statement on whether measurements were taken from distinct samples or whether the same sample was measured repeatedly
☒	☐	The statistical test(s) used AND whether they are one- or two-sided<br>*Only common tests should be described solely by name; describe more complex techniques in the Methods section.*
☒	☐	A description of all covariates tested
☒	☐	A description of any assumptions or corrections, such as tests of normality and adjustment for multiple comparisons
☐	☒	A full description of the statistical parameters including central tendency (e.g. means) or other basic estimates (e.g. regression coefficient) AND variation (e.g. standard deviation) or associated estimates of uncertainty (e.g. confidence intervals)
☒	☐	For null hypothesis testing, the test statistic (e.g. *F*, *t*, *r*) with confidence intervals, effect sizes, degrees of freedom and *P* value noted<br>*Give P values as exact values whenever suitable.*
☒	☐	For Bayesian analysis, information on the choice of priors and Markov chain Monte Carlo settings
☒	☐	For hierarchical and complex designs, identification of the appropriate level for tests and full reporting of outcomes
☒	☐	Estimates of effect sizes (e.g. Cohen's *d*, Pearson's *r*), indicating how they were calculated

Our web collection on statistics for biologists contains articles on many of the points above.

Software and code

Policy information about availability of computer code

Data collection

Software summary provided in software policy sheet. Not used for data collection, but included here for completeness:
SMART repository: DOI 10.5281/zenodo.10019463
SMART biological test cases: DOI 10.5281/zenodo.11268944

Data analysis

SMART relies on several other software packages. Unless otherwise noted, the most recent version of the package is currently supported. These are listed in the SMART repository but also included here for completeness.
- SMART uses FEniCS to assemble finite element matrices from the variational form. The current version of SMART uses the development version of SMART available as a Docker image that includes gmsh: ghcr.io/scientificcomputing/fenics-gmsh:2024-05-30.
- SMART uses PETSc4py to solve the resultant linear algebra systems.
- SMART uses pandas as an intermediate data structure to help organize and process models.
- SMART uses Pint for unit tracking and conversions.
- SMART uses matplotlib to generate plots in examples
- SMART uses sympy to allow users to input custom reactions and also to determine the appopriate solution techniques (e.g. testing for non-linearities).
- SMART uses numpy (>=v1.16.0, <v2.0) and scipy (>=v1.1.0) for general array manipulations and basic calculations.
- SMART uses tabulate to make ASCII tables.
- SMART uses termcolor for colored terminal output.

SMART has additional (optional) dependencies that are utilized for mesh processing in this work:
- Gmsh for mesh generation.
- meshio for mesh file type conversion.

All 3D renderings were performed using Paraview (v5.11.2)

For manuscripts utilizing custom algorithms or software that are central to the research but not yet described in published literature, software must be made available to editors and reviewers. We strongly encourage code deposition in a community repository (e.g. GitHub). See the Nature Portfolio guidelines for submitting code & software for further information.

Data

Policy information about availability of data

All manuscripts must include a data availability statement. This statement should provide the following information, where applicable:

- Accession codes, unique identifiers, or web links for publicly available datasets
- A description of any restrictions on data availability
- For clinical datasets or third party data, please ensure that the statement adheres to our policy

The results (average concentrations and other relevant outputs) from all of our simulations can be downloaded from our associated Zenodo repository https://zenodo.org/records/11252055. The data specifically included in each figure can be found in our source data Excel file.

External datasets were used for realistic subcellular geometries. All processed meshes used in our simulations are freely available in an associated Zenodo repository https://zenodo.org/records/10480304. The original sources are cited in the manuscript and also included here:

1. Dendritic spines: The spine imaging data used in this work are from Wu, Y.; Whiteus, C.; Xu, C. S.; Hayworth, K. J.; Weinberg, R. J.; Hess, H. F.; Camilli, P. D. Contacts between the Endoplasmic Reticulum and Other Membranes in Neurons. PNAS 2017, 114 (24), E4859–E4867. https://doi.org/10.1073/pnas.170107811
2. Calcium release unit (cardiomyocyte): Hoshijima, M. et al. CCDB:3603, MUS MUSCULUS, T-tubules, sarcoplasmic reticulum, myocyte, DOI: doi:10.7295/W9CCDB3603 (2004)
3. Mitochondrion: Mendelsohn, R. et al. Morphological principles of neuronal mitochondria. The Journal of Comparative Neurology 530, 886–902, DOI: 10.1002/cne.25254 (2022).

Human research participants

Policy information about studies involving human research participants and Sex and Gender in Research.

Reporting on sex and gender	n/a
Population characteristics	n/a
Recruitment	n/a
Ethics oversight	n/a

Note that full information on the approval of the study protocol must also be provided in the manuscript.

Field-specific reporting

Please select the one below that is the best fit for your research. If you are not sure, read the appropriate sections before making your selection.

☒ Life sciences ☐ Behavioural & social sciences ☐ Ecological, evolutionary & environmental sciences

For a reference copy of the document with all sections, see nature.com/documents/nr-reporting-summary-flat.pdf

Life sciences study design

All studies must disclose on these points even when the disclosure is negative.

Sample size	Timing was assessed over multiple runs in Supplementary Figure 2 to ensure consistent performance. We used a sample size of 5 for our two coarser meshes and a sample size of 3 for the finest mesh (due to increased computational time required). We determined these samples were sufficient given the consistency of timing outputs, with very small variance across runs.
Data exclusions	No data were excluded from our analyses.
Replication	We ran multiple simulations of dendritic spine calcium signaling (see sample size note above) to ensure consistency. All data can is readily replicated by downloading our code and running it locally.
Randomization	There were no experimental data included in this study for which randomization would apply.
Blinding	There were no experimental data included for which blinding might apply.

Reporting for specific materials, systems and methods

We require information from authors about some types of materials, experimental systems and methods used in many studies. Here, indicate whether each material, system or method listed is relevant to your study. If you are not sure if a list item applies to your research, read the appropriate section before selecting a response.

Materials & experimental systems

n/a	Involved in the study
☒	☐ Antibodies
☒	☐ Eukaryotic cell lines
☒	☐ Palaeontology and archaeology
☒	☐ Animals and other organisms
☒	☐ Clinical data
☒	☐ Dual use research of concern

Methods

n/a	Involved in the study
☒	☐ ChIP-seq
☒	☐ Flow cytometry
☒	☐ MRI-based neuroimaging
